# The clinical, economic, and patient‐centric burden of insomnia symptom severity in adults with major depressive disorder in the United States

**DOI:** 10.1002/brb3.3143

**Published:** 2023-07-12

**Authors:** Kruti Joshi, M. Janelle Cambron‐Mellott, Halley Costantino, Alanna Pfau, Manish K. Jha

**Affiliations:** ^1^ Janssen Scientific Affairs LLC Titusville New Jersey USA; ^2^ Cerner Enviza, an Oracle Company Kansas City Missouri USA; ^3^ Department of Psychiatry Icahn School of Medicine at Mount Sinai New York New York USA; ^4^ Center for Depression Research and Clinical Care, O'Donnell Brain Institute University of Texas Southwestern Medical Center Dallas Texas USA

**Keywords:** burden of illness, depression, insomnia, MDD

## Abstract

**Introduction:**

Insomnia is prevalent in adults with major depressive disorder (MDD) and is a key diagnostic criterion of MDD; however, little is understood about the burden of insomnia symptom severity in MDD. We evaluated the relationship between insomnia symptom severity and the clinical, economic, and patient‐centric burden among community‐dwelling individuals with MDD.

**Methods:**

Respondents with diagnosed depression who reported insomnia symptoms in the past 12 months (*N* = 4402) were identified from the 2019 United States National Health and Wellness Survey. Multivariable analyses assessed the association of Insomnia Severity Index (ISI) with health‐related outcomes while controlling for sociodemographic and health characteristics. Further analyses also controlled for depression severity (9‐item Patient Health Questionnaire).

**Results:**

Mean ISI score was 14.3 ± 5.6. Higher ISI was associated with greater depression severity (*r* = .51, *p* < .001). After adjustments, a one‐standard deviation (5.6‐point) increase in ISI score was significantly associated with higher depression (rate ratio [RR] = 1.36), anxiety (RR = 1.33) and daytime sleepiness (RR = 1.16) levels, more healthcare provider (RR = 1.13) and emergency room visits (RR = 1.31), hospitalizations (RR = 1.21), work productivity and activity impairment (RRs = 1.27 and 1.23, respectively), and poorer mental and physical health‐related quality of life (*β* = −3.853 and −1.999, respectively) (*p* < .001). These findings remained statistically significant when controlling for concurrent depression severity.

**Conclusion:**

In adults with MDD, greater insomnia symptom severity is associated with worse health‐related outcomes, which suggests the importance of addressing insomnia symptoms as a clinical target for treating MDD.

## Graphical abstract

Little is understood about the burden of insomnia symptom severity in adults with major depressive disorder (MDD). We evaluated the relationship between severity of insomnia symptoms and the clinical, economic, and patient‐centric burden among community‐dwelling individuals with MDD (N = 4,402) using data from the 2019 US National Health and Wellness Survey. In this population, greater insomnia symptom severity is associated with worse health‐related outcomes, which suggests the importance of addressing insomnia symptoms as a clinical target for treating MDD. Adjusted patient‐centric outcomes by insomnia symptom severity: (a) adjusted MCS, PCS, and EQ VAS scores by ISI score, and b) adjusted work productivity and activity impairment by ISI score

Note: Reference groups — Age: 46, Gender: female, Race: white, Ethnicity: non‐Hispanic, Marital Status: single, BMI: normal weight, Smoking Status: never smoker, Alcohol Use: less often than once a week, Insurance: Commercial, CCI: 0

Abbreviations: BMI, body mass index; CCI, Charlson comorbidity index; HCP, healthcare provider; ISI, Insomnia Severity Index; MCS, mental component summary; PCS, physical component summary; VAS, visual analogue scale

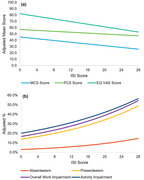

## 1 INTRODUCTION

Major depressive disorder (MDD) is a prevalent psychiatric condition and is often associated with significant societal burden (Santomauro et al., 2021). In the United States (US) alone, MDD accounts for the second most reported cause of disability in adults (American Psychiatric Association, 2022) and has created a greater overall burden than the economic impact of cancer and diabetes (Mrazek et al., 2014; US Burden of Disease Collaborators, 2013). The estimated lifetime prevalence of MDD in the US is 20.6% (Hasin et al., 2018).

The prevalence of MDD is higher among patient populations with sleep disorders (Mosko et al., 1989; Sivertsen et al., 2012), and sleep disturbances are common among those with clinical depression (Sivertsen et al., 2012; Yates et al., 2004). In particular, insomnia is considered a key diagnostic criterion of MDD (American Psychiatric Association, 2022). Insomnia symptoms are also a risk factor of new‐onset or recurrent depression in adults (Chang et al., 1997; Sivertsen et al., 2012; Weissman et al., 1997). Fang et al. (2019) concluded that treating sleep disorders before, during, and after episodes of MDD limited its recurrence and improved outcomes.

Although many research studies have documented the burden of experiencing insomnia, as a disorder or as insomnia symptoms (Bolge et al., 2009; Daley et al., 2009; Kyle et al., 2010), as well as the burden of MDD (Friedrich, 2017; Lépine & Briley, 2011; Stewart et al., 2003), few research studies have examined the burden of insomnia symptom severity among those with MDD. These few studies have mostly focused on the presence or absence of insomnia as a symptom and did not evaluate the association of insomnia symptom severity with health‐related outcomes among adults with MDD (Asche et al., 2010; Bolge et al., 2010). We identified one study that examined the relation between insomnia severity and health‐related quality of life (HRQoL) among an MDD population in Switzerland and found that higher insomnia symptom severity was associated with worse HRQoL (Jermann et al., 2021). However, that study examined only one aspect of burden, did not control for confounding variables, and was conducted with a clinical population (i.e., those currently seeking care for MDD) in a limited geographical area; the present study attempts to address these gaps. The objective of this study was to quantify the clinical, patient‐centric, and economic burden associated with the severity of insomnia symptoms among community‐dwelling adults who self‐reported a physician diagnosis of depression in the US (who may or may not currently be receiving care for depression), using a nationally representative data source.

## 2 MATERIALS AND METHODS

### 2.1 Data source and study design

Data for this retrospective, cross‐sectional analysis was obtained from the US 2019 National Health and Wellness Survey (NHWS), an internet‐based, patient‐reported, general population survey administered annually to approximately 75,000 adults ≥18 years old residing in the US. Potential respondents were recruited through an existing, general‐purpose (as opposed to healthcare‐specific) web‐based consumer panel who recruited members through opt‐in e‐mails, co‐registration with partners, e‐newsletter campaigns, banner placements, and affiliate networks. Quota sampling was used to ensure that the demographic composition of the NHWS sample was representative of the US adult population in terms of age, sex, and race.

### 2.2 Study sample

**FIGURE 1:**
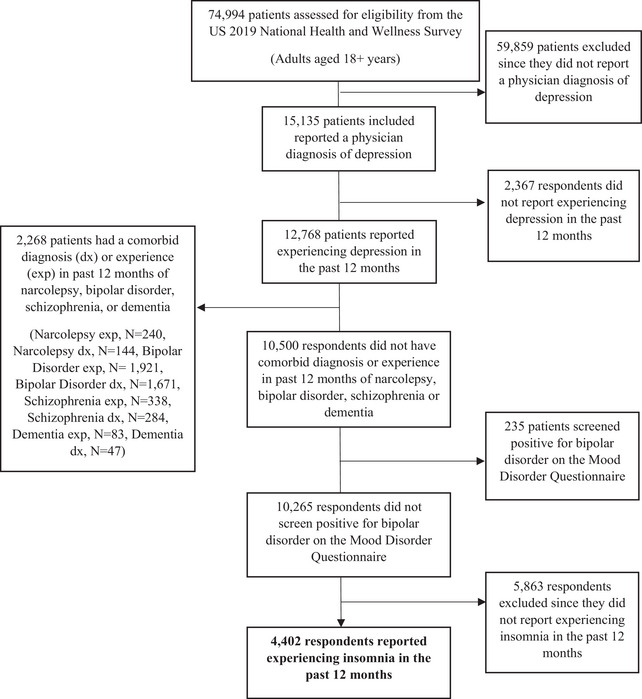
Study flowchart. dx, diagnosed; exp, experienced in last 12 months.

Respondents were included in this study if they self‐reported a physician diagnosis of depression and experienced both depression and insomnia in the past 12 months. Respondents were excluded if they self‐reported experiencing or being diagnosed with bipolar disorder, schizophrenia, dementia, or narcolepsy, or screened positive for bipolar disorder on the Mood Disorder Questionnaire. The study flowchart is found in Figure 1.

### 2.3 Measures

Sociodemographic characteristics included age, sex, race, ethnicity, marital status, and insurance coverage. Health characteristics included body mass index (BMI), smoking status, alcohol use, and the Charlson Comorbidity Index (CCI) (Quan et al., 2011).

#### 2.3.1 Independent variable

Insomnia symptom severity was assessed by the Insomnia Severity Index (ISI) which measures the perception of both nocturnal and diurnal symptoms of insomnia (Morin et al., 2011). Respondents rated seven items on a 5‐point scale from 0 (none) to 4 (very severe); items were summed to form the measure of severity (range: 0–28).

#### 2.3.2 Clinical burden

The 9‐item Patient Health Questionnaire (PHQ‐9) (Kroenke et al., 2001) measured depression severity. Respondents indicated the extent to which they experienced nine symptoms of depression in the last 2 weeks on a scale of 0, not at all, to 3, nearly every day. Items were summed to form a total score (range: 0–27).

Anxiety severity was assessed with the 7‐item General Anxiety Disorder (GAD‐7) questionnaire. Respondents indicated the extent to which they experienced seven symptoms of anxiety in the last 2 weeks using a 4‐point scale ranging from 0 (not at all) to 3 (nearly every day) (Spitzer et al., 2006). Items were summed to form a total score ranging from 0 to 21.

The Medication Adherence Reasons Scale (MAR‐Scale) evaluated the frequency of and the reasons for medication nonadherence among those taking a daily oral prescription medication for depression (Unni et al., 2014). The measure consisted of one global item where respondents indicated the number of days the medication was taken in the last 7 days (range: 0–7), and 19 items frequently cited by patients as common reasons for medication nonadherence. Respondents rated these 19 items on an 8‐point scale from 0 to 7 days; the items were summed to form a total score ranging from 0 to 133 with higher scores indicating a higher complexity of nonadherence with more reasons and more days.

The Epworth Sleepiness Scale (ESS) measured the propensity for sleep in an individual's daily life (Johns, 1991). Respondents indicated the likelihood of falling asleep in eight different scenarios based a 4‐point scale going from 0 (would never doze) to 4 (high chance of dozing). Scores were summed up to form a total range of 0–24 with higher scores indicating greater sleepiness.

Healthcare resource utilization (HCRU) included the number of visits to traditional healthcare providers, psychiatrists, psychologists, and emergency rooms (ERs) and the number of hospitalizations in the past 6 months.

#### 2.3.3 Patient‐centric burden

Patient‐centric burden included both HRQoL and work productivity and activity impairment (WPAI) instruments. HRQoL measures included the Medical Outcomes Study 36‐Item Short Form Survey Instrument Version 2 (SF‐36v2) (Maruish, 2011) and the 5‐level EQ‐5D version (EQ‐5D‐5L) (Herdman et al., 2011). The SF‐36v2 is a 36‐item, multipurpose, generic health status instrument that maps onto eight health domains. Four domains are used to form a mental component summary (MCS): vitality, role functioning (emotional), mental health, and social functioning, and another four domains are used to form the physical component summary (PCS): general health, physical functioning, bodily pain, and physical role limitations. Each summary score in the SF‐36v2 is normed to a mean of 50 with a standard deviation (SD) of 10 for the general US population; higher scores indicate a better health status (Maruish, 2011). The short‐form six‐dimensions (SF‐6D) utility scores, a preference‐based single index measure for health using general population values, were derived from the SF‐36v2 and were used in this study (Brazier et al., 2002). The measure is considered a continuous outcome scored on a 0.29–1.00 scale; higher scores indicate better overall general health (Walters & Brazier, 2002).

The EQ‐5D‐5L consists of a descriptive system and a visual analogue scale (EQ VAS). The descriptive system comprised five dimensions, including mobility, self‐care, usual activities, pain/discomfort, and anxiety/depression, in which respondents indicate the amount of problems with each dimension on a 5‐point rating scale. Index scores were calculated by mapping the five‐level descriptive system onto the three‐level valuation set using the mapping approach developed by van Hout et al. (2012). Index scores typically range from less than 0–1, where less than 0 indicates a health state worse than death, 0 indicates a health state equivalent to death, and 1 indicates a health state equivalent to perfect health. The EQ VAS asked respondents to indicate their self‐rated health on a line having endpoints of 0 (worst imaginable health state) and 10 (best imaginable health state).

The WPAI—General Health questionnaire is a 6‐item validated instrument that assessed four domains with a 1‐week recall period: absenteeism, presenteeism, overall work productivity impairment, and activity impairment. Higher values, given in percentages (%), indicate greater impairment due to the respondent's health (Reilly et al., 1993). All participants completed the activity impairment section, whereas only participants that were currently employed were eligible to answer the questions that assessed work‐related activities.

#### 2.3.4 Economic burden

Direct costs were estimated by multiplying the number of healthcare provider visits, ER visits, and hospitalizations in the past 6 months by 2, multiplying this number by the unit cost for each type of visit obtained from the Medical Expenditure Panel Survey data (Agency for Healthcare Research and Quality, 2018.) and then inflated to 2020 medical care costs (Federal Reserve Bank of St Louis, 2021). Indirect costs were calculated using the human capital method (Onukwugha et al., 2016) by applying estimated age‐, sex‐, and race‐adjusted wages provided by the US Bureau of Labor Statistics (2020) to WPAI‐reported absenteeism and presenteeism estimates.

### 2.4 Statistical analyses

Sociodemographic and health characteristics were summarized to provide a comprehensive overview of respondents. Continuous variables were summarized as means with SD, and categorical variables were reported as frequencies and percentages (%). Pearson's correlation analysis was conducted to examine the linear association of insomnia symptom severity (ISI score) with depression severity (PHQ‐9 score).

In the multivariable analyses, generalized linear models (GLMs) were used to assess the association of insomnia symptom severity (ISI score) with clinical, patient‐centric, and economic outcomes after adjusting for the following covariates: age, sex, race, ethnicity, CCI, marital status, BMI, smoking status, alcohol use, and insurance type. GLMs specifying a normal distribution were used for the MCS, PCS, SF‐6D, and EQ‐5D variables; parameter estimates with standard errors and *p* values were reported. As all other outcomes represented a count of the amount of time (e.g., PHQ‐9 and HCRU), GLMs specifying a negative binomial distribution were used for all other measures; parameter estimates with standard errors, adjusted rate ratios (RRs) with 95% confidence intervals (CIs) were reported. The RR is the amount of change in the outcome variable associated with a 1‐point increase in the predictor variable. Two‐tailed tests were considered statistically significant when *p* < .05. Parameter estimates and RR were adjusted to account for a 1‐SD increase in ISI scores. This was done to demonstrate the change in outcomes expected given a clinically meaningful increase in ISI score. Lastly, given the potential association of insomnia symptoms with greater depression severity, sensitivity analyses were conducted to evaluate whether the relation between ISI and outcomes held when adjusting for depression severity; therefore, the multivariable analyses described above were rerun with PHQ‐9 score added to the models as an additional covariate.

## 3 RESULTS

In total, 4402 adults with MDD who experienced insomnia symptoms in the past 12 months were included in the analyses (Figure 1). Respondent characteristics are shown in Table 1. Most patients were female (71.8%), White (77.8%), and non‐Hispanic (88.1%). The mean age was 45.1 ± 15.8 years, and the mean CCI score was 0.38 ± 1.00. The mean PHQ‐9 score was 11.3 ± 6.7 (range: 0–27), and the mean ISI score was 14.3 ± 5.6 (range: 0–28) (Table 1). Higher ISI scores were associated with greater severity of depression (*r* = .51, *p* < .001).

**TABLE 1:** Sociodemographic and health characteristics of adults with major depressive disorder (MDD) experiencing insomnia symptoms in the United States, *N* = 4402.

Characteristic		
Age	*Mean (SD)*	45.1 (15.8)
	*Median (min—max)*	46 (18–85)
Sex, *N* (%)	*Male*	1242 (28.2)
	*Female*	3160 (71.8)
Race, *N* (%)	*White*	3424 (77.8)
	*Black/African American*	383 (8.7)
	*Asian*	142 (3.2)
	*Some other race or origin/multi‐race*	453 (10.3)
Ethnicity, *N* (%)	*Hispanic*	523 (11.9)
	*Not Hispanic*	3879 (88.1)
Marital status, *N* (%)	*Married/living with a partner*	1986 (45.1)
	*Single/not married/divorced/separated/widowed/decline to answer*	2416 (54.9)
Insurance type, *N* (%)	*Commercially insured*	1967 (44.7)
	*Medicaid*	607 (13.8)
	*Medicare*	918 (20.9)
	*Other type of insurance*	447 (10.2)
	*Not insured*	463 (10.5)
CCI score	*Mean (SD)*	0.38 (1.00)
	*Median (Min–Max)*	0 (0–22)
BMI, *N* (%)	*Underweight (<18.5)*	94 (2.1)
	*Normal weight (18.5 to <25)*	1247 (28.3)
	*Overweight (25 to <30)*	1103 (25.1)
	*Obese (30 or greater)*	1830 (41.6)
	*Unknown*	128 (2.9)
Smoking status, *N* (%)	*Current smoker*	929 (21.1)
	*Former smoker*	1328 (30.2)
	*Never smoked*	2145 (48.7)
Alcohol use, *N* (%)	*Daily*	220 (5.0)
	*4–6 times a week; 2–3 times a week; once a week*	1146 (26.0)
	*Less often than once a week*	3036 (69.0)
PHQ‐9 score	*Mean (SD)*	11.3 (6.7)
	*Median (Q1–Q3)*	10 (6–16)
ISI score	*Mean (SD)*	14.3 (5.6)
	*Median (Q1–Q3)*	14 (10–18)

Abbreviations: BMI, body mass index; CCI, Charlson comorbidity index; ISI, Insomnia Severity Index; max, maximum; min, minimum; PHQ‐9, 9‐item Patient Health Questionnaire; Q1, first quartile; Q3, third quartile; SD, standard deviation.

### 3.1 The clinical burden of insomnia symptom severity

**FIGURE 2:**
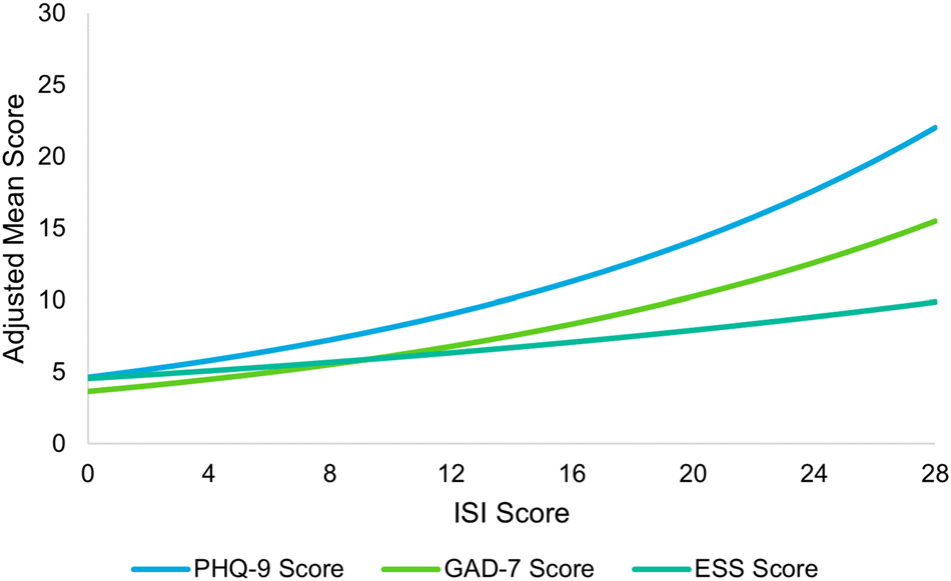
Adjusted clinical outcomes by insomnia symptom severity. *Note*: Reference groups—age: 46, gender: female, race: white, ethnicity: non‐Hispanic, marital status: single, body mass index (BMI): normal weight, smoking status: never smoker, alcohol use: less often than once a week, insurance: commercial, Charlson Comorbidity Index (CCI): 0. ESS, Epworth Sleepiness Scale; GAD‐7, 7 item General Anxiety Disorder scale; ISI, Insomnia Severity Index; PHQ‐9, 9‐item Patient Health Questionnaire.

Results of the multivariable analysis indicated that a higher severity of insomnia symptoms was associated with greater depression, anxiety, and daytime sleepiness (Figure 2). More specifically, when keeping other predictors constant, each 1‐SD (5.6 points) higher ISI score was associated with a 1.36 times higher PHQ‐9 score (*p* < .001), a 1.33 times higher GAD‐7 score (*p* < .001), and a 1.16 times higher ESS score (*p* < .001) (Table 2). In addition, higher insomnia symptom severity was associated with lower medication adherence and higher complexity of medication nonadherence, such that each 1‐SD (5.6 points) higher ISI score was associated with a 0.98 times lower MAR‐Scale global item (*p* = .023) and a 1.29 times higher MAR‐Scale score (*p* < .001), which indicates a higher complexity of nonadherence with more reasons and more days (Table 2).

**TABLE 2:** Results of the multivariable analyses examining the association of insomnia symptom severity with clinical, patient‐centric, and economic outcomes.

	ISI score (1‐point increase)				1‐SD increase (5.6 points)	
	*N*	*β* (SE)	RR (95% CI)	*p*	*β* (SE)	RR (95% CI)
Screening tools^a^						
*PHQ‐9 score*	4402	.055 (.002)	1.06 (1.05–1.06)	<.001	.308 (.009)	1.36 (1.34–1.39)
*GAD‐7 score*	4325	.051 (.002)	1.05 (1.05–1.06)	<.001	.286 (.011)	1.33 (1.30–1.36)
*MAR‐scale global item*	2474	−.003 (.001)	.997 (.995–1.00)	.023	−.017 (.007)	.98 (.97–1.00)
*MAR‐scale score*	2474	.045 (.010)	1.05 (1.03–1.07)	<.001	.252 (.055)	1.29 (1.15–1.43)
*ESS score*	4325	.027 (.002)	1.03 (1.02–1.03)	<.001	.151 (.011)	1.16 (1.14–1.19)
HCRU^a^ in the past 6 months						
*Healthcare provider visits*	4402	.022 (.003)	1.02 (1.02–1.03)	<.001	.123 (.018)	1.13 (1.09–1.17)
*Psychologist/therapist visits*	4402	.025 (.008)	1.03 (1.01–1.04)	.003	.140 (.046)	1.15 (1.05–1.26)
*Psychiatrist visits*	4402	.022 (.008)	1.02 (1.01–1.04)	.006	.123 (.045)	1.13 (1.04–1.24)
*Emergency room visits*	4402	.048 (.007)	1.05 (1.04–1.06)	<.001	.269 (.038)	1.31 (1.21–1.41)
*Hospitalizations*	4402	.034 (.009)	1.03 (1.02–1.05)	<.001	.190 (.053)	1.21 (1.09–1.34)
HRQoL^b^						
*MCS score*	4402	−.688 (.028)	.	<.001	−3.853 (.157)	.
*PCS score*	4402	−.357 (.026)	.	<.001	−1.999 (.144)	.
*SF‐6D utility score*	4402	−.007 (.000)	.	<.001	−.039 (.002)	.
*EQ‐5D index score*	4402	−.010 (.000)	.	<.001	−.056 (.002)	.
*EQ VAS score*	4402	−1.035 (.060)	.	<.001	−5.796 (.337)	.
WPAI^a^						
*Absenteeism (mean %)*	2181	.057 (.008)	1.06 (1.04–1.08)	<.001	.319 (.043)	1.38 (1.27–1.50)
*Presenteeism (mean %)*	2130	.045 (.003)	1.05 (1.04–1.05)	<.001	.252 (.018)	1.29 (1.24–1.33)
*Overall work productivity impairment (mean %)*	2181	.042 (.003)	1.04 (1.04–1.05)	<.001	.235 (.017)	1.27 (1.22–1.31)
*Activity impairment (mean %)*	4402	.037 (.002)	1.04 (1.03–1.04)	<.001	.207 (.010)	1.23 (1.21–1.26)
Economic burden^a^						
*Total direct medical costs*	4402	.030 (.006)	1.03 (1.02–1.04)	<.001	.168 (.031)	1.18 (1.11–1.26)
*Total indirect costs*	2272	.047 (.004)	1.05 (1.04–1.06)	<.001	.263 (.022)	1.30 (1.25–1.36)

*Note*: controlling for age, sex, race, ethnicity, marital status, BMI, smoking status, alcohol use, insurance type, CCI score.

Abbreviations: BMI, body mass index; CCI, Charlson comorbidity index; CI, confidence interval; ESS, Epworth Sleepiness Scale; GAD‐7, 7‐item General Anxiety Disorder scale; HCRU, healthcare resource utilization; HRQoL, health‐related quality of life; ISI, Insomnia Severity Index; MAR, Medication Adherence Reasons; MCS, mental component summary; PCS, physical component summary; PHQ‐9, 9‐item Patient Health Questionnaire; RR, rate ratio; SD, standard deviation; SE, standard error; SF‐6D, short‐form six‐dimensions; VAS, visual analog scale; WPAI, work productivity and activity impairment questionnaire.

^a^ Interpretation: For each 1‐point/1‐SD (5.6 points) increase in ISI score 〈the outcome〉 is 〈exp(*β*)〉 times higher, keeping other predictors constant.

^b^ Interpretation: For each 1‐point/1‐SD (5.6 points) increase in ISI score 〈the outcome〉 changes by an average of <*β*>, keeping other predictors constant.

Greater HCRU was associated with a higher severity of insomnia symptoms (Figure S1). Each 1‐SD (5.6 points) higher ISI score was associated with 1.13 times more healthcare provider visits (*p* < .001), 1.15 times more psychologist/therapist visits (*p* = .003), 1.13 times more psychiatrist visits (*p* = .006), 1.31 times more ER visits (*p* < .001), and 1.21 times more hospitalizations (*p* < .001) in the past 6 months (Table 2).

### 3.2 The patient‐centric burden of insomnia symptom severity

**FIGURE 3:**
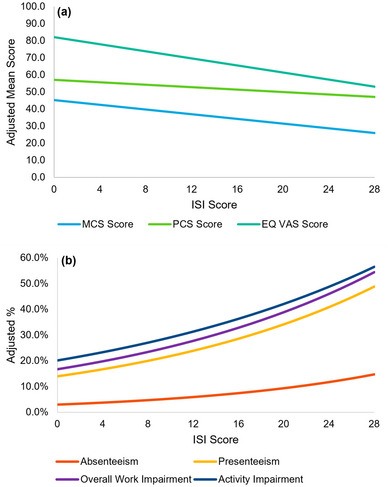
Adjusted patient‐centric outcomes by insomnia symptom severity: (a) adjusted mental component summary (MCS), physical component summary (PCS), and EQ visual analogue scale (VAS) scores by Insomnia Severity Index (ISI) score, and (b) adjusted work productivity and activity impairment by ISI score. *Note*: Reference groups—age: 46, gender: female, race: white, ethnicity: non‐Hispanic, marital status: single, body mass index (BMI): normal weight, smoking status: never smoker, alcohol use: less often than once a week, insurance: commercial, Charlson Comorbidity Index (CCI): 0.

Higher severity of insomnia symptoms was associated with worse HRQoL (Figure 3a and Figure S2). A 1‐SD (5.6 points) higher ISI score was associated with a 3.85 lower MCS score (*p <* .001), a 2.00 lower PCS score (*p* < .001), and a 0.039 lower SF‐6D utility score (*p* < .001). Additionally, a 1‐SD higher ISI score was associated with a 0.056 lower EQ‐5D index score (*p* < .001) and a 5.80 lower EQ VAS score (*p* < .001) (Table 2).

In addition, higher severity of insomnia symptoms was associated with greater impairment of work productivity and activity (Figure 3b). Specifically, a 1‐SD (5.6 points) higher ISI score was associated with a 1.38 times higher rate of absenteeism (*p* < .001), a 1.29 times higher rate of presenteeism (*p* < .001), a 1.27 times higher rate of overall work productivity impairment (*p* < .001), and a 1.23 times higher rate of nonwork‐related activity impairment (*p* < .001) (Table 2).

### 3.3 The economic burden of insomnia symptom severity

**FIGURE 4:**
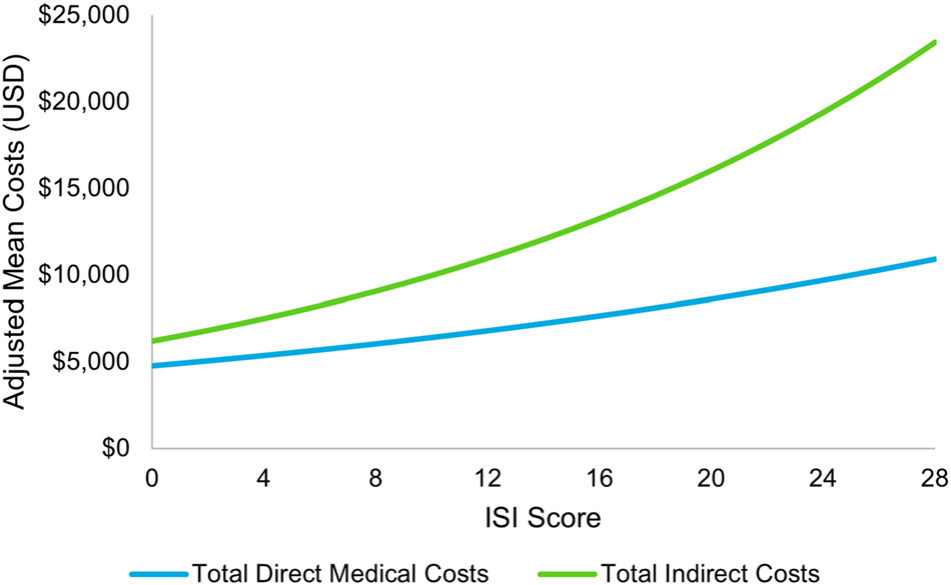
Adjusted economic outcomes by insomnia symptom severity. *Note*: Reference groups—age: 46, gender: female, race: white, ethnicity: non‐Hispanic, marital status: single, body mass index (BMI): normal weight, smoking status: never smoker, alcohol use: less often than once a week, insurance: commercial, Charlson Comorbidity Index (CCI): 0.

Higher severity of insomnia symptoms was associated with higher direct medical costs and indirect costs in terms of lost wages due to missed time from work (Figure 4). In particular, a 1‐SD (5.6 points) higher ISI score was associated with 1.18 times higher total direct medical costs (*p* < .001) and 1.30 times higher total indirect costs (*p* < .001) (Table 2).

### 3.4 Analyses after adjusting for severity of depressive symptoms

In the multivariable analyses that were adjusted for the severity of depressive symptoms, higher severity of insomnia symptoms was still significantly associated with greater anxiety and daytime sleepiness, greater complexity of medication nonadherence, more healthcare provider visits, ER visits, and hospitalizations, poorer HRQoL, greater absenteeism, presenteeism, WPAI, and higher direct medical costs and indirect costs (Table 3).

**TABLE 3:** Results of multivariable analyses examining the association of insomnia symptom severity with health‐related outcomes after adjusting for depression severity.

	ISI score (1‐point increase)				1‐SD increase (5.6 points)	
	*N*	*β* (SE)	RR (95% CI)	*p*	*β* (SE)	RR (95% CI)
Screening tools^a^						
*GAD‐7 score*	4325	.016 (.002)	1.012 (1.01–1.02)	<.001	.090 (.011)	1.09 (1.07–1.12)
*MAR‐scale global item*	2474	−.001 (.001)	.999 (.997–1.002)	.663	−.006 (.008)	.99 (.98–1.01)
*MAR‐scale score*	2474	.028 (.012)	1.03 (1.01–1.05)	.018	.157 (.067)	1.17 (1.03–1.33)
*ESS score*	4325	.020 (.002)	1.02 (1.02–1.02)	<.001	.112 (.012)	1.12 (1.09–1.15)
HCRU in the last 6 months^a^						
*Healthcare provider visits*	4402	.016 (.004)	1.02 (1.01–1.02)	<.001	.090 (.021)	1.09 (1.05–1.14)
*Psychologist/therapist visits*	4402	−.002 (.010)	.998 (.98–1.02)	.850	−.011 (.054)	.99 (.89–1.10)
*Psychiatrist visits*	4402	.008 (.009)	1.01 (.99–1.03)	.384	.045 (.052)	1.05 (.95–1.16)
*Emergency room visits*	4402	.040 (.008)	1.04 (1.03–1.06)	<.001	.224 (.044)	1.25 (1.15–1.36)
*Hospitalizations*	4402	.026 (.011)	1.03 (1.01–1.05)	.016	.146 (.060)	1.16 (1.03–1.30)
HRQoL^b^						
*MCS score*	4402	−.083 (.026)		.002	−.465 (.148)	
*PCS score*	4402	−.281 (.030)		<.001	−1.574 (.165)	
*SF‐6D utility score*	4402	−.002 (.000)		<.001	−.011 (.002)	
*EQ‐5D index score*	4402	−.004 (.000)		<.001	−.022 (.002)	
*EQ VAS score*	4402	−.472 (.067)		<.001	−2.643 (.376)	
WPAI^a^						
*Absenteeism (mean %)*	2181	.036 (.009)	1.04 (1.02–1.06)	<.001	.202 (.050)	1.22 (1.11–1.35)
*Presenteeism (mean %)*	2130	.026 (.004)	1.03 (1.02–1.03)	<.001	.146 (.021)	1.16 (1.11–1.21)
*Overall work productivity impairment (mean %)*	2181	.024 (.004)	1.03 (1.02–1.03)	<.001	.134 (.020)	1.14 (1.10–1.19)
*Activity impairment (mean %)*	4402	.019 (.002)	1.02 (1.02–1.02)	<.001	.106 (.012)	1.11 (1.09–1.14)
Economic burden^a^						
*Total direct medical costs*	4402	.023 (.006)	1.02 (1.01–1.04)	<.001	.129 (.036)	1.14 (1.06–1.22)
*Total indirect costs*	2272	.028 (.005)	1.03 (1.02–1.04)	<.001	.157 (.025)	1.17 (1.11–1.23)

*Note*: controlling for age, sex, race, ethnicity, marital status, BMI, smoking status, alcohol use, insurance type, CCI score, PHQ‐9 score.

Abbreviations: BMI, body mass index; CCI, Charlson comorbidity index; CI, confidence interval; ESS, Epworth Sleepiness Scale; GAD‐7, 7 item General Anxiety Disorder scale; HCRU, healthcare resource utilization; HRQoL, health‐related quality of life; ISI, Insomnia Severity Index; MAR, Medication Adherence Reasons; MCS, mental component summary; PCS, physical component summary; PHQ‐9, 9‐item Patient Health Questionnaire; RR, rate ratio; SD, standard deviation; SE, standard error; SF‐6D, short‐form six‐dimensions; VAS, visual analog scale; WPAI, work productivity and activity impairment questionnaire.

^a^ Interpretation: For each 1‐point/1‐SD (5.6 points) increase in ISI score 〈the outcome〉 is 〈exp(*β*)〉 times higher, keeping other predictors constant.

^b^ Interpretation: For each 1‐point/1‐SD (5.6 points) increase in ISI score 〈the outcome〉 changes by an average of 〈*β*〉, keeping other predictors constant.

## 4 DISCUSSION

To our knowledge, this was the first large‐scale study to evaluate the relationship between severity of insomnia symptoms among community‐dwelling adults with MDD in the US and clinical, patient‐centric, and economic outcomes. We found that the burden of insomnia was greater at higher levels of insomnia symptoms which corresponded with greater severity of depression, anxiety, and daytime sleepiness, increased HCRU, poorer HRQoL, greater impairment of work productivity and nonprofessional activities, and greater direct and indirect costs.

In the current study, higher insomnia severity was associated with greater patient‐centric burden among adults with MDD. Similar results were found in Bolge et al. (2010) where adults with MDD who experienced chronic sleep maintenance insomnia had greater impairment in work productivity and activity as well as poorer mental and physical HRQoL. However, it is worth noting that the study only focused on the presence versus absence of insomnia characterized by nighttime awakenings that had moderate to severe impact on respondents’ personal and professional life (Bolge et al., 2010). Our results are also supported by another study that showed that higher insomnia levels were associated with poorer HRQoL among a clinical sample of patients with MDD in Switzerland (Jermann et al., 2021).

In this present study, higher insomnia symptom severity was associated with greater HCRU and direct costs. These findings are consistent with a large managed care claims database that observed a higher number of outpatient visits and higher direct costs among patients with MDD and insomnia compared to matched patients with MDD and no insomnia (Asche et al., 2010). Our findings are also congruent with a study using 2006 NHWS data that found more ER visits and hospitalizations among adults with diagnosed depression who were experiencing insomnia symptoms compared to adults diagnosed with depression who were not experiencing insomnia (Bolge et al., 2010).

We calculated a 1‐SD increase in ISI score to estimate the added burden a clinically meaningful increase in ISI score would have on health‐related outcomes. This equated to a 5.6‐point increase in ISI score, which is similar to the minimally importance difference identified by Yang et al. (2009) and provides support for the use of 1‐SD change as a measure of clinical meaningfulness.

Notably, this study demonstrated that although insomnia symptom severity and depression severity are highly correlated, insomnia symptom severity is uniquely associated with worse outcomes. When controlling for depression severity, we found that higher insomnia symptom severity was associated with greater clinical, patient‐centric, and economic burden on most of the health‐related outcomes included in the study. However, the parameter estimates were reduced when accounting for depression severity. These results may suggest that depression severity partially mediates the relation between insomnia symptom severity and health‐related outcomes. It is also possible that depression severity is a moderator of this relationship, and thus, the association of insomnia symptom severity and health‐related outcomes may differ at different levels of depression severity. Future research is needed to understand the exact relation among insomnia symptoms severity, depression symptom severity, and health‐related outcomes. One approach could be to conduct a stratified analysis of the relationship between ISI score and outcomes after stratifying by depression severity level cut‐points.

One may also argue that because the PHQ‐9 has a sleep‐related item and the ISI has a question on distress, the two measures overlap and measure similar constructs. This could have led to spurious results when entering depression severity into the model. However, given that the correlation between the two instruments was 0.51, it is unlikely that multicollinearity was an issue in the multivariable analyses. Given that the relations between insomnia symptom severity and the outcomes were in the same direction, just reduced, when PHQ‐9 was entered as a covariate, suggests further that multicollinearity was not an issue. As both the EQ‐5D and MCS contain items regarding depression, it is more likely that PHQ‐9 has a stronger association with those outcomes due to measuring similar constructs than does ISI, and thus by entering PHQ‐9 as a covariate, the relation between ISI and these outcomes was attenuated.

Currently, pharmacological treatment of adults with MDD and insomnia is challenging. Many of the available antidepressants affect sleep regulation to some degree; for example, insomnia is a frequently reported side effect of selective serotonin reuptake inhibitors (Spigset, 1999). Thus, treatment with antidepressants that affect sleep regulation may exacerbate existing sleep disturbance. Indeed, in one study, 16% of patients reported worsening in insomnia during the first 6 weeks of antidepressant treatment, which in turn was associated with lower likelihood of long‐term remission (Jha et al., 2018). Low‐dose sedating antidepressants, such as mirtazapine or trazodone, may be efficacious in treating insomnia but are not adequate for treating depression (Schutte‐Rodin et al., 2008). Trazodone has been particularly cited as one of the least efficacious antidepressants (Cipriani et al., 2018). Given the complexity of treating insomnia in MDD, the lack of evidence‐based treatments for insomnia symptoms among the MDD population and the association of insomnia with relapse and recurrence of depressive episodes (Chang et al., 1997; Sivertsen et al., 2012; Weissman et al., 1997) treatment for insomnia is currently an unmet need in clinical practice. Further, our findings demonstrate that the treatment of insomnia symptoms among patients with MDD is an unmet need and suggests that more effective treatments targeting both insomnia and depressive symptoms are warranted.

It may be worth noting that the timing of the 2019 NHWS was conducted before the implications of the COVID‐19 pandemic, and this may have affected the impact of insomnia symptom severity in adults with MDD. Many of the mental and psychological health concerns generated as a result of the global outbreak may further indicate a need to provide care in terms of treatment for insomnia symptoms and MDD not only for the general population but also for healthcare workers (Cénat et al., 2021; Wu et al., 2021).

The key strengths of this study were the use of a survey designed to match the US general population in terms of age, sex, and race, and the use of validated instruments to assess insomnia symptom severity and health‐related outcomes. However, this study was not without limitations. There was potential bias between the respondents that took the online survey and those who did not; for example, our study may have underrepresented the elderly population or those without access to the internet.

Self‐reported data may have produced bias as the information provided could not be confirmed independently and may have involved inaccurate recall and/or false reporting. For example, the diagnosis of depression was based on self‐report and was not confirmed by a clinician diagnosis or a structured diagnostic instrument. Moreover, although measured variables were accounted for in the statistical modeling, there may have been the possibility of unmeasured variables that influenced both insomnia symptom severity and the health‐related outcomes. Lastly, due to the nature of cross‐sectional studies, no causal relationship between insomnia symptom severity in MDD and the health‐related outcomes could be made.

## 5 CONCLUSION

Among adults with MDD, higher levels of insomnia are associated with greater clinical, economic, and patient‐centric burden. Even after the severity of depression was controlled, higher levels of insomnia were associated with worse health‐related outcomes. Our findings emphasize the need for MDD treatments that can clinically target the insomnia symptoms, in addition to the depressive symptoms in order to improve overall health‐related outcomes in patients with MDD.

## CONFLICT OF INTEREST STATEMENT

K. Joshi and A. Pfau are employees of Janssen Scientific Affairs, LLC and also own stock in Johnson & Johnson. M.J. Cambron‐Mellott and H. Costantino are employees of Cerner Enviza, which received funding from Janssen Scientific. Affairs, LLC, to conduct and report on the study. M.K. Jha has received contract research grants from Acadia Pharmaceuticals, Neurocrine Biosciences, Navitor/Supernus, and Janssen Research & Development; an educational grant to serve as section editor of the Psychiatry & Behavioral Health Learning Network; consultant fees from Eleusis Therapeutics US, Inc., Janssen Global Services, Worldwide Clinical Trials/Eliem Therapeutics, and Guidepoint; and honoraria from the North American Center for Continuing Medical Education, WebMD/Medscape, and Global Medical Education. Dr. Jha did not receive any honoraria/payments for this study.

### PEER REVIEW

The peer review history for this article is available at https://publons.com/publon/10.1002/brb3.3143.

## Supporting information

Figure S1 Adjusted healthcare resource utilization by insomnia symptom severity: (a) number of HCP visits in the past 6 months by ISI score; (b) number of psychologist/therapist and psychiatrist visits in the past 6 months by ISI score; and (c) number of ER visits and hospitalizations in the past 6 months by ISI score.

Figure S2 Adjusted patient‐centric outcomes by insomnia symptom severity: SF‐6D utility and EQ‐5D index scores by ISI score.

## Data Availability

Research data are not shared.
