# Supplementary material for: The clinical, economic, and patient‐centric burden of insomnia symptom severity in adults with major depressive disorder in the United States
Source: Brain Behav. 2023 Jul 12;13(8):e3143. doi: 10.1002/brb3.3143 (PMC10454259; doi:10.1002/brb3.3143)
Supplement: Supplementary file 1 — Figure S1 Adjusted healthcare resource utilization by insomnia symptom severity: (a) number of HCP visits in the past 6 months by ISI score; (b) number of psychologist/therapist and psychiatrist visits in the past 6 months by ISI score; and (c) number of ER visits and hospitalizations in the past 6 months by ISI score. [file BRB3-13-e3143-s001.pdf]

Figure S1. Adjusted healthcare resource utilization by insomnia symptoms severity: (a) number of HCP visits in the past 6 months; (b) number of psychologist/therapist and psychiatrist visits in past 6 months by ISI score; and c) number of ER visits and hospitalizations in past 6 months by ISI score.

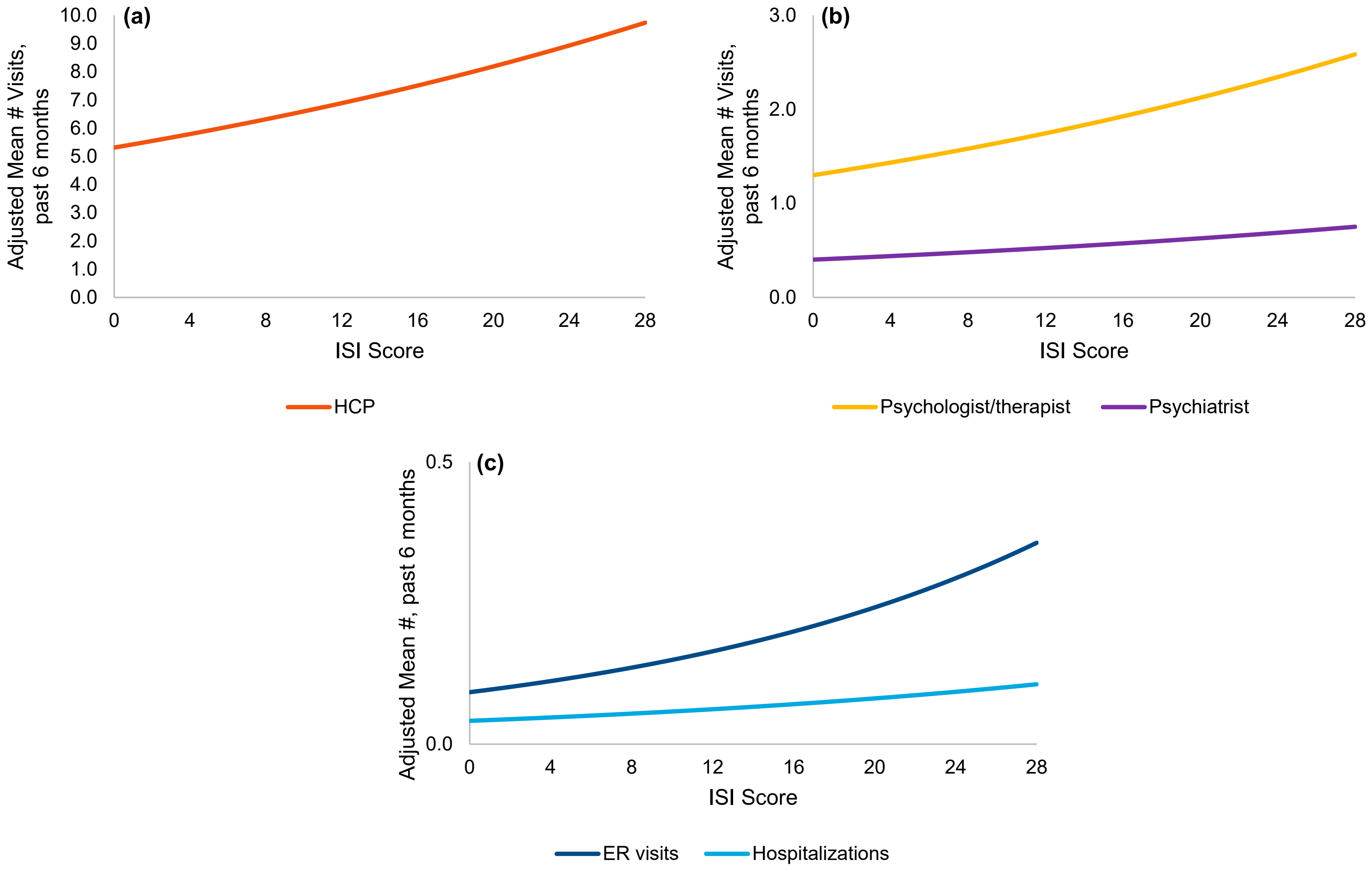

Note: Reference groups -- Age: 46, Gender: female, Race: white, Ethnicity: non-Hispanic, Marital Status: single, BMI: normal weight, Smoking Status: never smoker, Alcohol Use: less often than once a week, Insurance: Commercial, CCI: 0.

Abbreviations: BMI, body mass index; CCI, Charlson comorbidity index; ER, emergency room; HCP, healthcare provider; ISI, Insomnia Severity Index
