# Supplementary material for: The clinical, economic, and patient‐centric burden of insomnia symptom severity in adults with major depressive disorder in the United States
Source: Brain Behav. 2023 Jul 12;13(8):e3143. doi: 10.1002/brb3.3143 (PMC10454259; doi:10.1002/brb3.3143)
Supplement: Supplementary file 2 — Figure S2 Adjusted patient‐centric outcomes by insomnia symptom severity: SF‐6D utility and EQ‐5D index scores by ISI score. [file BRB3-13-e3143-s002.pdf]

Figure S2. Adjusted patient-centric outcomes by insomnia symptom severity: SF-6D utility and EQ-5D index scores by ISI score

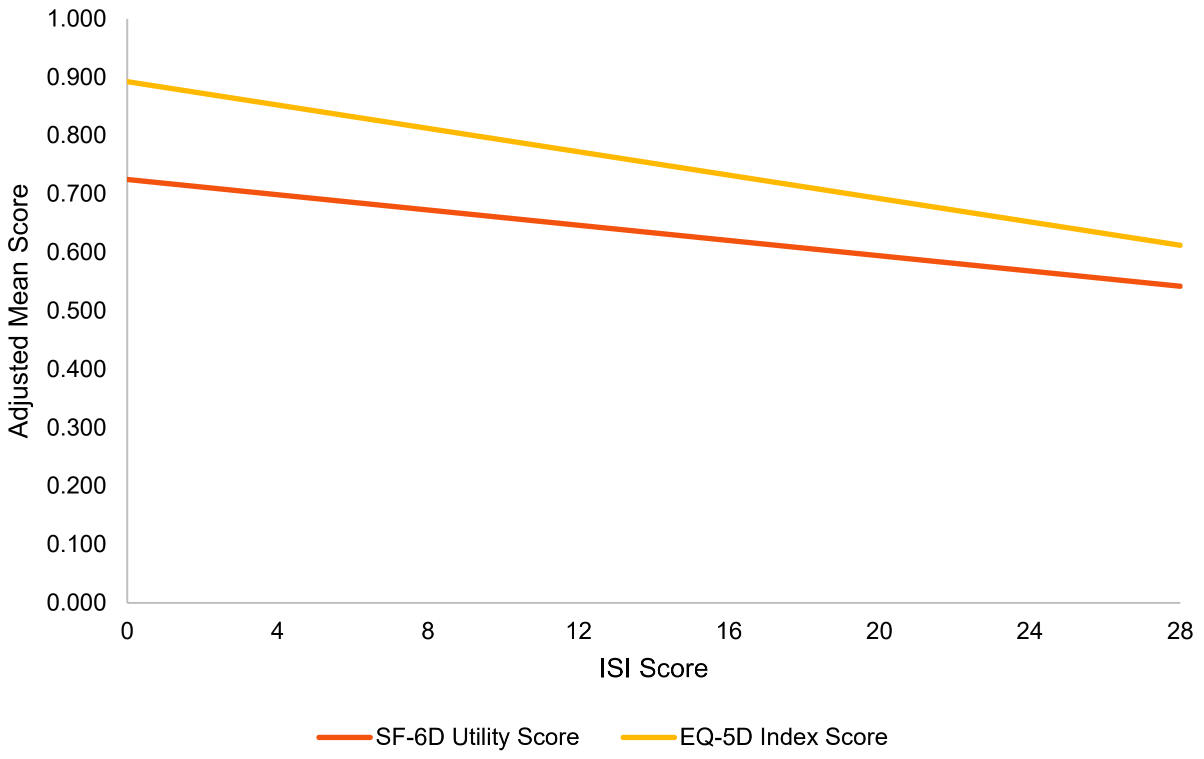

Note: Reference groups -- Age: 46, Gender: female, Race: white, Ethnicity: non-Hispanic, Marital Status: single, BMI: normal weight, Smoking Status: never smoker, Alcohol Use: less often than once a week, Insurance: Commercial, CCI: 0.

Abbreviations: BMI, body mass index; CCI, Charlson comorbidity index; ISI, Insomnia Severity Index; SF-6D, Short-Form-Six-Dimensions
